# Case Study: Free Lunch Meals Provision during the Remote Learning Conditions

**DOI:** 10.3390/nu13020605

**Published:** 2021-02-12

**Authors:** Ilze Beitane, Zanda Kruma, Tatjana Kince, Martins Sabovics, Sandra Iriste, Sandra Muizniece-Brasava, Jekaterina Bujaka, Sintija Strode, Inga Ciprovica

**Affiliations:** 1 Department of Nutrition, Faculty of Food Technology, Latvia University of Life Sciences and Technologies, Jelgava LV-3004, Latvia; sandra.iriste@llu.lv (S.I.); Jekaterina.bujaka@llu.lv (J.B.); 2 Department of Food Technology, Faculty of Food Technology, Latvia University of Life Sciences and Technologies, Jelgava LV-3004, Latvia; zanda.kruma@llu.lv (Z.K.); tatjana.kince@llu.lv (T.K.); martins.sabovics@llu.lv (M.S.); sandra.muizniece@llu.lv (S.M.-B.); sintija.strode@llu.lv (S.S.); inga.ciprovica@llu.lv (I.C.)

**Keywords:** food packs, nutritional recommendations, recipes, nutrients and energy

## Abstract

School meals for grade 1 to 4 pupils in Latvia are financed by the government, but with the onset of the COVID-19 pandemic in March 2020, and following the remote learning process, there were problems related to the delivery of these meals for pupils. The current situation in Latvia has been exacerbated again due to the spread of the pandemic; there is a great necessity to find well-thought-out solutions to ensure school lunches outside the school. The aim of this study was to develop recommendation-based one-week food packs for grade 1 to 4 pupils, providing the necessary amount of nutrients and energy. Four food packs were designed to provide five-day lunch meals for pupils, preparing a warm lunch at home. Protein, fat, saturated fatty acids, carbohydrates, sugar, dietary fiber, sodium, salt and calcium content of meals were analyzed according to standard methods. During the project, the most appropriate solution for food packs was explored. The four designed food packs will provide support to municipalities, because the composition of food packs complies with the nutrition and energy value regulation and does not exceed the planned budget. Parents will receive the developed recipe book in addition to a one-week food pack.

## 1. Introduction

With the onset of the COVID-19 pandemic in March 2020, the school learning process was provided remotely and a number of issues and challenges were raised in relation to free lunch meals. Latvian municipalities addressed this by creating food packs for one week, taking into account the nutritional recommendations of the Ministry of Health. Approved nutritional recommendations in the Cabinet of Ministers Regulation No. 172 of the Republic of Latvia [1] require that approximately 35% of total energy should be covered at lunch, corresponding to the percentage of protein, fat, and carbohydrates. In order to assist municipalities in designing one-week food packs, the Ministry of Health developed new recommendations on the provision of food packs to pupils during an emergency situation [2]. The recommendations state that the one-week food pack must contain at least 900 g of fruit and vegetables, at least 600 g of milk and dairy products, at least 300 g of processed grain products, at least 300 g of potatoes, at least 500 g of protein-rich products, and 90 g of fats such as oil or butter. However, various aspects, such as the provision of nutrients and energy, were not taken into account by municipalities in the designing of the food pack’s composition. The provision of nutrients is essential for children’s physical and mental development, emphasizing that 35% of the daily energy needs should be taken with lunch.

Adequate intake of fruit and vegetables is one of the basic components of a healthy diet. World Health Organization (WHO) [3] data confirm that low fruit and vegetable intake increases the risk of mortality and morbidity, such as gastrointestinal cancer, ischemic heart disease, and stroke. The results of a study by the Centre for Disease Control and Prevention [4] on the health of Latvian pupils showed that the intake of fruit and vegetables among pupils is insufficient: only 26.8% of pupils eat at least one portion of fruit per day, and 27.2% eat at least one portion of vegetables per day, while 55% of 11-year-old children do not consume at least one portion of either fruit or vegetables per day. In addition, the WHO recommendations [5] state that at least five portions of fruit and vegetables should be consumed per day, meaning that each meal includes at least one portion of fruit or/and vegetables. The food packs designed by municipalities contained a poor amount of fruit and vegetables. In turn, the study by Nasirzadeh et al. [6] confirmed a strong correlation between the availability and intake of fruit and vegetables. By including fruit and vegetables in food packs, it is possible to enhance their consumption among pupils. In addition, it is essential that municipal schools implement the European Union (EU)-supported program “School milk and fruit”, which can increase daily fruit consumption.

It is recommended to include milk and dairy products in food packs, and at the same time, the selected products must be stored at room temperature, so municipalities choose ultra-high temperature (UHT) milk. Milk and dairy products are a part of a healthy diet of pupils and have a beneficial effect on child’s growth, because milk contains high-quality proteins, fats, carbohydrates, vitamins, minerals, and insulin-like growth factor–1 [7,8,9]. A study analyzing milk intake among children aged 6–59 months confirmed a strong association between milk consumption and children’s growth, as milk consumption reduced the risk of underweight [9]. In addition, milk contains an optimal ratio of calcium and phosphorus, which is supplemented by the concentration of magnesium in milk, all together ensuring the development of the skeleton in children [10]. The study on health behavior among the Latvian adult population in 2018 [11] found that 54.9% of respondents aged 15 to 24 do not drink milk at all. In order to increase the number of pupils who drink milk, Latvian schools are implementing the EU-supported program “School milk and fruit”. A total of 243,000 pupils in Latvia received milk in the 2019/2020 school year, representing 87% of the target group [12]. Especially in the context of remote learning, the implementation of this program and the provision of nutrient-dense products in the daily nutrition of pupils are essential.

The food packs should include products containing complex carbohydrates, so municipalities are more likely to choose bread, pasta, oatmeal, and wheat flour. Grain products and potatoes form the fundamental components of the Latvian diet, providing more than half of the necessary energy intake. In addition, emphasis is placed on whole grain products, as their intake is associated with beneficial health effects, reducing the risk of oncological and cardiovascular diseases [13]. Whole grain products were rarely included in food packs. World experience has shown that it is important to offer these products to children from an early age in order to promote the intake of whole grain products among the population [14]. Taking into account the potato consumption traditions of the Latvian population, a certain amount of potatoes should be included in food packs. The benefit of potatoes is the concentration of vitamin C and B_1_, flavonoids and phenols [15]. According to Food and Agriculture Organization Statistics (FAOSTAT) data [16], potato consumption in Latvia reached 124 kg per capita in 2017, ranking in third place among 155 countries. The data confirm the popularity of potatoes among the Latvian population and their inclusion in the recommendations.

The inclusion of protein-rich products in pupils’ diet, including food packs, is of particular importance. It is important to provide sufficient high-quality protein in children’s diet to ensure their adequate growth [17]. Special attention should be paid to the restriction of essential amino acids such as lysine and tryptophan [18], so the inclusion of animal origin proteins in children’s diet is recommended [19]. Studies confirm that legumes can also be a good source of high-quality protein; raw legumes have a low bioavailability, which could be improved by processing [20]. The protein content required for children’s diet can be achieved by mixing animal- and plant-origin proteins.

The quantity and quality of fat play an important role in children’s diet. According to the recommended energy and nutrient intake for Latvian population, developed by the Ministry of Health, fat comprises 30–35% of the total daily energy for children aged 4 to 18 [21]. The WHO recommendations [5] clearly state that saturated fatty acids should constitute than 10% of the total energy intake, while trans-fatty acids should comprise less than 1% of the total energy intake. Considering that children eat fish at a low rate, eggs should be included in their diet as a source of long chain *n*-3 PUFA [22].

Considering that the situation in Latvia has been exacerbated again due to the spread of coronavirus, there is a great necessity to find well-thought-out solutions to ensure the delivery of school lunches outside school and to propose the model for food pack provision. The aim of this study was to develop recommendation-based one-week food packs for grade 1 to 4 pupils, providing the necessary amount of nutrients and energy.

## 2. Materials and Methods

The study was implemented in the period from July to November 2020 and was divided into several stages: (1) analysis of recommendations and legislation acts; (2) selection of food products for food packs; (3) development of recipes from selected products to prepare healthy lunches for pupils throughout the week; (4) calculation of nutritional value and energy based on information from product labels and food products databases; (5) testing the recipes in practice; (6) performing the analysis of nutritional components and value for produced lunch meals; and (7) analysis of obtained results. During the implementation of the research, one-week food packs were adjusted and improved in various stages of the study.

The following conditions were taken into account in the process of one-week food pack design: (1) the provision of energy and nutrients for grade 1 to 4 pupils aged 7 to 11 years, in accordance with the recommendations of the Ministry of Health [2,21] and the Cabinet of Ministers Regulation No. 172 [1], which prescribes that the energy value is between 2058 and 3150 kJ, the protein content is from 12 to 28 g, the fat content is from 16 to 29 g, and the carbohydrates content is from 55 to 113 g for lunch. (2) Dietary diversity in line with healthy eating recommendations [5]. (3) Recommended quantities for certain product groups. (4) Limited costs of one-week food packs (EUR 7.10 per pack), as the government provides municipalities with financing to cover the benefit-related free school meals. (5) The storage conditions of the selected products must correspond to room temperature to ensure that the quality of the products is maintained during distribution. (6) The weight/volume of the products offered for sale, so that municipalities can design food packs using packaging size offered by producers. (7) Food recipes from the products included in the food packs.

In view of the above conditions, four one-week food packs were developed, analyzing the protein, fat, saturated fatty acids, carbohydrates, sugar, dietary fiber, sodium, salt, and calcium content in the developed meals. The fat content was detected by the Soxhlet extraction method. The protein concentration was analyzed by Kjeldahl titrimetric method. Fatty acids were measured according to ISO 12966-1:2014, ISO 12966-2:2017, and ISO 12966-4:2015 standard methodology. Individual sugars were analyzed by the enzymatic-spectrophotometric method. Carbohydrates and energy values were calculated according to Regulation No. 1169/2011 of the European Parliament and the Council of 25 October 2011 methodology. The sodium concentration was analyzed by Mohr’s titrimetric method and the salt concentration was calculated on the basis of data on sodium. Dietary fiber was analyzed by the Association of Official Agricultural Chemists (AOAC) 991.43:1994 method. Calcium was measured by the atomic emission spectrophotometry method with inductively coupled plasma.

The summary of nutritional composition is shown in the heat map and the calculation is based on z-scores. All data were processed using Microsoft Excel 2013.

## 3. Results

As a result of the study, four one-week food packs were designed to provide a five-day lunch meal for pupils outside the school, preparing warm lunches at home in line with developed recipes. The list of products included in one-week food packs is summarized in Table 1. 

The principle of food diversity was considered in the design of one-week food packs, selecting a variety of processed grain products (rice, pasta, pearl-barley, etc.), protein-dense products (beef, chicken, pork, legumes, eggs, etc.), as well as vegetables in various fixed amounts.

In addition to the one-week food pack, pupils were provided with UHT milk (200 mL per day) and apples according to the EU-supported program “School milk and fruit”. Consequently, milk and apples were used in the development of recipes, leaving some of the apples for fresh intake.

Five-day lunch recipes were developed for each food pack (Table 2), taking into account the recommendations for the provision of protein, fat, carbohydrates, and energy for pupils in lunch. In addition, the content of sugar, saturated fatty acids, dietary fiber, salt, calcium, and sodium was analyzed. The obtained data are presented in Table 3, Table 4, Table 5 and Table 6.

Taking into account the eating traditions of Latvian population and the high consumption of bread in Latvia, which was 52.64 kg per capita in 2018 [23], 35 g of rye bread was included in almost every lunch.

In order to promote the intake of vegetables, according to WHO recommendations [3,5], fresh-cut vegetables (carrots, cucumbers, tomatoes, and bell peppers) were included as snacks before or after lunch in a few days, as the availability of fruit and vegetables has a positive effect on their intake [6].

The designed lunch of food pack No. 1 products covered the protein, fat, and carbohydrate needs for grade 1 to 4 pupils, while energy intake was slightly below the recommendations for a few days.

The nutrients and energy indicated in recommendations were achieved in meals prepared from food pack No. 2 products, except on Friday, where the content of protein and energy was slightly lower compared to needs of pupils, as specified in Cabinet Regulation No. 172. 

The meals prepared from products of food pack No. 3 were in line with the nutritional value, with the exception of the protein content at meals on Thursday, as well as the energy provision in meals on Tuesday and Friday.

The meals from food pack No. 4 were characterized by a lower fat content on Friday compared to the recommendations; the content of other nutrients was in line with the recommendations.

**Figure 1:**
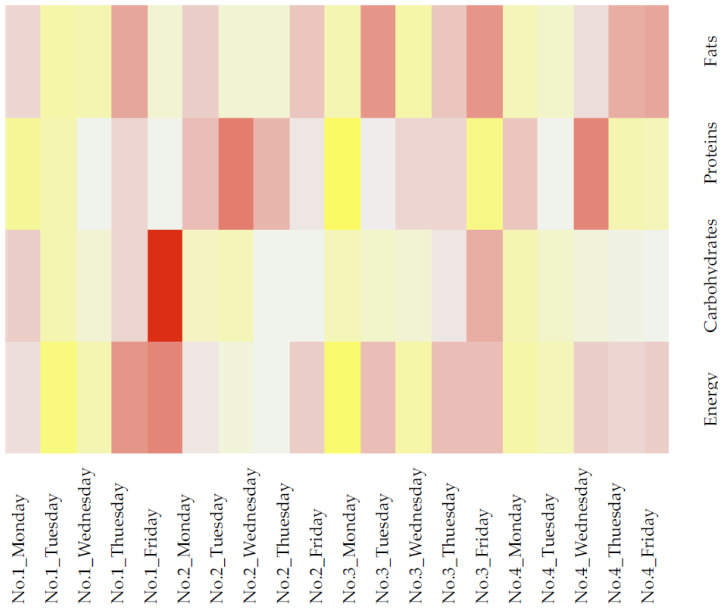
Heat map of nutrients in lunch meals. Abbreviations used in figure: No. 1 to No. 4–food packs.

The summary of the nutritional composition for macronutrients (carbohydrates, fats, proteins) and energy is presented in the heat map (Figure 1) and the calculation is based on z-scores. Yellow squares present lower level of nutrient on the current day, whites describe the medium level, while reddish colors indicate the highest levels comparing all days. Generally, fat variation is higher, showing lower and higher values at all periods, whereas carbohydrates are at constant levels in all lunch meals. 

Each lunch meal was developed in accordance with the guidelines, but it was also important to assess the variation of nutrients in food packs from a weekly perspective (Table 7).

The total protein and fat variation was higher and ranged from 19.56 to 32.64%, and 16.83 to 31.35%, respectively. More invariable nutrients in current lunch meals included carbohydrates, with an average variation of 13.07%. The energy of lunch meals varied the least, where that of food pack No. 4 was only 7.07%.

## 4. Discussion

The most serious challenges in designing food packs were the provision of nutrients and energy, because it is difficult to balance the products in food packs with the nutrient and energy needs in meals. The first highlighted issue during the study was that the provision of the recommended nutrient needs did not mean meeting energy needs—for instance, meals prepared from products of food pack No. 1 on Tuesday and Wednesday. In this case, the recommendations should be reviewed in order to avoid contradiction between nutrient intake and energy need.

Assessing the amount of protein in days, the content of protein at lunch depended on whether meat/fish/eggs were included or not. On the days when the abovementioned products were included, the protein content was higher than the recommended minimum. The challenge was to provide the protein content for lunch when excluding meat/fish/eggs; there were positive examples (food pack No. 1 on Monday and Friday), but in two cases, the goal was not achieved, e.g., one-week food pack No. 2 on Friday and one-week food pack No. 3 on Thursday. In order to achieve the goal, the portion size should be increased, which was not acceptable, considering that recipes were developed for pupils aged 7 to 11 years. This confirms the conclusions of the literature [19] that it is necessary to include animal-origin proteins in children’s diet in order to achieve the desired result with regard to the protein content in small quantities.

The provision of fat in lunch meals was not a problem, except for food pack No. 4 on Friday, where the fat content was lower than recommended. Greater attention had to be paid to ensure that the fat recommendation was not exceeded on certain days when canned meat was included—for instance, pasta with pork (food pack No. 3 on Monday). In addition, the concentration of saturated fatty acids (SFA) was analyzed in order to assess the quality of fat and compliance with the general recommendations of healthy nutrition [5,21], the concentration of SFA should not exceed 10% of total energy. The content of SFA in lunch meals ranged from 3.17 to 7.92 g, which is in line with recommendations, except for two days, where SFA content was 12.24 g (food pack No. 1 on Monday) and 12.80 g (food pack No. 3 on Monday), which is close to 50% of the total fat content at lunch. In the first case, the increased SFA content was mainly caused by butter and sour cream, and in the second case it was caused by canned pork. In order to change elevated SFA content, it would be necessary to reduce the amount of these products, but care should be taken to ensure that the total content of fat is not lower than recommended.

The recommended content of carbohydrates was provided in all lunches, but it approached the lower limit of the recommendations. The carbohydrate content varied from 55.00 to 92.40 g at lunch; however, more than half of all lunches had carbohydrate content ranging from 55.00 to 65.00 g. The nutrition calculation did not include an apple if it was not intended to be included in the recipe. Assuming that the pupils eat the fresh apples, it would increase the content of carbohydrates in pupil diet during the day. In addition, the sugar content of lunch meals was analyzed as one of the initiatives of WHO and the Ministry of Health of Latvia is to reduce sugar consumption among the population, recommending that the sugar content should not exceed 10% of the total energy [5,21]. The results of the study show that the sugar content in lunch meals is a sufficiently critical issue. The lowest sugar content at lunch was 7.82 g, which would be in line with recommendations, while the highest sugar content was 27.81 g, according to the nutrient and energy intake of the Latvian population, the average recommended energy intake for children aged 7 to 10 is 7766 kJ [21], which means that sugar content should not exceed 46 g per day. This, in turn, leads to the conclusion that the sugar content of one-week food pack No. 3 on Monday covers 60% of the permitted sugar per day. The reason for the high content of sugar for this particular lunch is the apple milkshake. In general, higher sugar content was achieved in the lunches where a dessert was included.

The dietary fiber content at lunch depends on the included products, such as recipes with canned beans, oatmeal, rye bread, etc., where dietary fiber content is higher. This was in line with the findings of other studies that wholegrain products (50%), legumes (30–40%), fruit and vegetables (16%) cover the total dietary fiber intake of humans [24]. Depending on the content of dietary fiber, all lunch meals could be divided in two groups: the first group has a relatively low dietary fiber content—up to 8.28 g—and the second group has a high dietary fiber content—between 11.49 and 19.20 g, based on the research data that the recommended content of dietary fiber for children is 19–31 g per day [25].

No salt was added during the meal cooking process, therefore sodium was determined in the meal and the obtained data of sodium were used to calculate the salt concentration. It should be noted that canned products such as meat, fish, and beans contain salt that increases the salt content in meals. Given that the storage conditions for all products in food packs must correspond to room temperature, it was not possible to include fresh meat/fish or their products, and therefore the only choice was canned meat/fish. Different canned meat/fish was used in each product pack and the results of the study confirm that the highest salt concentration was achieved in recipes for canned beef (food pack No. 1) and canned fish/beans (food pack No. 4). Overall, the salt concentration at lunch ranged from 0.36 to 3.11 g. In order to reduce salt content in recipes, it would be necessary, together with producers who would be ready to produce products for one-week food packs according to governmental procurement, to assess the possibility of reducing the salt content in canned meat/fish/beans. According to Cabinet Regulation No. 172, meat and fish products included in pupils’ diet must comply with certain requirements, i.e., the salt content must not exceed 1.25 g per 100 g of meat product or 1.5 g per 100 g of fish product [1].

The content of calcium in the meals is sufficiently high, except for two meals on Monday and Thursday in food pack No. 1. In ten meals, the calcium content ranges from 100 to 200 mg, and in eight meals it is above 200 mg. In addition to the one-week food pack, pupils are also given milk, 200 mL per day. Assuming that the pupil drinks 200 mL of milk every day, which is about 240 mg of calcium [26], than the total calcium content for lunch could be nearly 400–500 mg, which would be 50–60% of the recommended calcium dietary allowance for children aged 7 to 11 years.

## 5. Conclusions

The four designed one-week food packs are in accordance with the nutritional guidelines and the planned budget of Latvia government and the possible diversity of selected products (e.g., as high protein sources in each one-week pack were selected canned pork, beef or poultry). The results of the current study will provide support for municipalities to cover benefits-related free school meals at home; for parents, because they will also receive recipe book in addition to one-week food pack; and for entrepreneurs, because they will be able to participate in public procurements by adjusting the weight/volume of the product pack according to the requirements of one-week food packs.

## Figures and Tables

**Table 1:** The product list of one-week food pack.

Food Groups	One-Week Food Packs			
	No. 1	No. 2	No. 3	No. 4
Potatoes	Potatoes	Potatoes	Potatoes	Potatoes
Fruit and vegetables	Red beet Cabbage Tomato Green peas Onion Carrot Apple	Carrot Cabbage Cucumber Bell pepper Onion Apple	Carrot Red beet Chinese cabbage Tomato Cucumber Onion Raisin Apple	Carrot Cucumber Bell pepper Onion Apple
Grain processing products	Buckwheat Pasta Millet Rye bread	Rice Oatmeal Pearl-barley Rye bread	Buckwheat Pasta Oatmeal Rye bread	Rice Pearl-barley Oatmeal Rye bread
Protein dense products	Canned beef Canned white beans	Canned fish Eggs	Canned pork Canned butter beans Eggs	Canned chicken Canned beans Eggs
Milk and dairy products	Sour cream Milk	Sour cream Milk	Sour cream Milk	Sour cream Milk

**Table 2:** Five-day lunch meals list of 4 one-week food packs.

Week Days	Food Packs			
	No. 1	No. 2	No. 3	No. 4
Monday	Millet-carrot scones with sour cream Red beet salad Bread	Cabbage soup Bread Omelet Oatmeal dessert	Pasta with pork Fresh vegetables snacks Milkshake	Risotto with meat Fresh vegetables snacks Oven fried apples
Tuesday	Beef soup Rye bread Apple keel	Vegetable risotto with fish Fresh vegetables snacks Apple milkshake	Red beet soup with sour cream Rye bread In oven fried apples	Potatoes with vegetables and meat Bean salad Bread
Wednesday	Mashed potatoes with meat sauce Tomato salad Rye bread	Fish soup with sour cream Bread Rice-apple pie	Buckwheat with meat sauce Red beet salad Rye bread	Potatoes soup with sour cream Rye bread Fresh vegetables snacks Rice pudding
Thursday	Boiled buckwheat with meat sauce Red beet and peas salad Rye bread	Fried eggs with potatoes Cucumber-bell pepper salad Oatmeal biscuits	Potato pancakes with sour cream Bean-cabbage salad Rye bread	Pearl-barley porridge with meat Fresh vegetables snacks Rye bread Apple keel
Friday	White bean soup with sour creamRye bread Pasta with vegetables	Pear-barley puree with cabbage and sour cream Bell pepper-cucumber salad Rye bread	Butter bean pie with potatoes Tomato-cucumber-Chinese cabbage salad Rye bread	Red bean soup with sour cream Rye bread In oven fried apples with oatmeal

**Table 3:** Nutritional value and energy of five-day lunch meals prepared from food pack No. 1.

Nutrients	Recommendations	Week Days				
		Monday	Tuesday	Wednesday	Thursday	Friday
Protein, g	12–28	12.16	14.01	17.59	20.13	17.75
Fat, g	16–29	23.89	16.11	17.31	27.71	19.57
SFA, g	−	12.24	4.45	3.89	7.42	5.39
Carbohydrates, g	55–113	69.09	55.01	59.52	67.77	92.64
Sugar, g	−	16.31	17.05	9.20	7.82	10.96
Dietary fiber, g	−	7.49	5.4	19.20	12.21	11.8
Sodium, g	2 *	0.34	0.80	0.84	0.63	0.59
Salt, g	5 *	0.86	2.01	2.09	1.60	1.48
Calcium, mg	800 *	94.95	112.59	150.99	55.00	184.62
Energy, kJ	2058–3150	2265	1769	1951	2520	2601

* Recommended dietary allowance [21]. SFA, saturated fatty acids.

**Table 4:** Nutritional value and energy of five-day lunch meals prepared from food pack No. 2.

Nutrients	Recommendations	Week Days				
		Monday	Tuesday	Wednesday	Thursday	Friday
Protein, g	12–28	21.78	25.75	21.96	18.64	9.02
Fat, g	16–29	24.44	19.66	19.87	25.51	16.94
SFA, g	−	4.57	5.04	5.38	3.17	4.47
Carbohydrates, g	55–113	56.95	55.61	62.84	62.94	55.49
Sugar, g	−	17.37	15.65	14.36	13.25	9.91
Dietary fiber, g	−	5.80	6.82	2.1	13.16	3.18
Sodium, g	2 *	0.41	0.50	0.66	0.14	0.36
Salt, g	5 *	1.04	1.27	1.65	0.36	0.89
Calcium, mg	800 *	204.55	378.31	175.62	138.07	147.20
Energy, kJ	2058–3150	2243	2111	2177	2331	1723

* Recommended dietary allowance [21]. SFA, saturated fatty acids.

**Table 5:** Nutritional value and energy of five-day lunch meals prepared from food pack No. 3.

Nutrients	Recommendations	Week Days				
		Monday	Tuesday	Wednesday	Thursday	Friday
Protein, g	12–28	18.44	19.79	19.80	10.83	21.21
Fat, g	16–29	29.00	16.08	25.10	28.93	17.37
SFA, g	−	12.80	6.01	7.92	5.43	5.73
Carbohydrates, g	55–113	57.91	59.10	65.38	73.84	55.00
Sugar, g	−	27.81	24.27	8.14	9.45	10.22
Dietary fiber, g	−	3.78	4.95	8.28	11.49	15.39
Sodium, g	2 *	0.66	0.75	0.91	0.34	0.51
Salt, g	5 *	1.66	1.86	2.27	0.85	1.28
Calcium, mg	800 *	165.21	179.35	290.12	214.26	234.61
Energy, kJ	2058–3150	2371	1936	2377	2366	1938

* Recommended dietary allowance [21]. SFA, saturated fatty acids.

**Table 6:** Nutritional value and energy of five-day lunch meals prepared from food pack No. 4.

Nutrients	Recommendations	Week Days				
		Monday	Tuesday	Wednesday	Thursday	Friday
Protein, g	12–28	17.41	25.20	13.84	14.08	19.44
Fat, g	16–29	18.94	23.56	27.16	27.69	11.27
SFA, g	−	5.14	6.07	6.55	5.47	4.40
Carbohydrates, g	55–113	58.66	60.47	61.72	62.94	78.77
Sugar, g	−	11.25	10.57	13.03	26.33	22.85
Dietary fiber, g	−	6.08	12.39	7.23	7.86	19.20
Sodium, g	2 *	0.95	1.24	0.92	1.02	0.59
Salt, g	5 *	2.38	3.11	2.29	2.55	1.47
Calcium, mg	800 *	124.32	295.6	213.42	254.49	179.79
Energy, kJ	2058–3150	1994	2328	2289	2334	2087

* Recommended dietary allowance [21]. SFA, saturated fatty acids.

**Table 7:** Variation coefficient of nutrients in food packs.

Samples	Variation Coefficient, %			
	Protein	Fat	Carbohydrates	Energy
Food pack No. 1	19.56	23.04	21.13	16.12
Food pack No. 2	32.64	16.83	6.48	11.09
Food pack No. 3	22.95	26.70	12.06	10.83
Food pack No. 4	25.91	31.35	12.60	7.07
Total	25.26	24.48	13.07	11.28

## Data Availability

Not applicable.
